# One Year Outcomes After Percutaneous Coronary Intervention in Diabetics With Stable Ischemic Heart Disease: A Single-Center Comparative Study

**DOI:** 10.7759/cureus.12731

**Published:** 2021-01-16

**Authors:** Muhammad Adil, Imran Khan, Zair Hassan, Syed A Habib, Muhammad S Jibran, Tariq Nawaz

**Affiliations:** 1 Cardiology, Lady Reading Hospital, Peshawar, PAK; 2 Internal Medicine, Hayyatabad Medical Complex, Peshawar, PAK

**Keywords:** diabetes, stable ischemic heart disease, one year outcomes

## Abstract

Introduction

Coronary artery disease is the leading cause of death not only in Pakistan but also worldwide. Coronary artery disease is prevalent in diabetes and is the major cause of morbidity and mortality. This study aims at comparing the long-term outcomes of patients with and without diabetes undergoing percutaneous coronary interventions (PCI) in in a tertiary care hospital.

Methods

This is a prospective study including 200 patients undergoing PCI for stable ischemic heart disease. All the patients were followed up over three, six months, and then over one year for major outcomes, including death, nonfatal myocardial infarction, and revascularization, including target vessel revascularization (TVR), and target lesion revascularization (TLR), as well as the outcome of a major adverse cardiovascular event (MACE).

Results

The mean age (standard deviation) of the non-diabetic with stable ischemic heart disease (SIHD) patients was higher (57.4±8.9 years) than diabetes mellitus (DM) patients. All baseline characteristics were not statistically significant between the two groups. Triple vessel disease prevalence was more in DM than in the non-DM patients with SIHD, although it was not statistically significant. The number of stents implanted per patient (2.8±0.7 vs 1.9±0.8) was more in DM patients than in non-DM patients with SIHD. In-hospital adverse outcomes, including death due to cardiovascular causes, periprocedural myocardial infarction, hyperacute stent thrombosis, and bleeding complications, were insignificant between the two groups. Contrast-induced nephropathy was more prevalent in diabetics with SIHD. Although one-year major adverse cardiovascular outcomes were common in the diabetic group, these were statistically insignificant.

Conclusion

PCI for complex lesions in stable ischemic heart disease, both with and without diabetes, is associated with favorable in-hospital and long-term outcomes with regards to MACE and ischemia-driven revascularization.

## Introduction

Coronary artery disease (CAD) is the leading cause of death not only in the developed world but also in the Indo-Pak subcontinent [1, 2]. Percutaneous coronary intervention (PCI) is a primary and common treatment for CAD. PCI has shown to have symptomatic benefits but survival benefits have not been proved. On the contrary, studies have shown increased survival benefits of both PCI in acute ST-segment elevation myocardial infarction (STEMI) [1]. PCI has also shown both early and long-term survival benefits in non-STEMI (NSTEMI) [2, 3]. However, there are questions regarding the long-term survival benefits of stable ischemic heart disease (SIHD). Diabetes mellitus (DM) is a major global health issue nowadays and the world faces a serious challenge as its prevalence and incidence continue to grow day by day. The prevalence of CAD in DM patients is a major cause of morbidity and mortality, and more than 80% of DM deaths occur in low- and middle-income countries [4, 5]. DM patients are more exposed to multivessel coronary disease compared to non-DM due to rapid atherosclerosis, coronary plaques, and endothelial injury [4-6]. DM patients undergoing coronary artery revascularization poses a challenge because of the increased risk of adverse outcomes after coronary artery bypass grafting (CABG) or PCI [7, 8]. The currently published guidelines endorse CABG over PCI in DM patients and multivessel coronary artery disease [9, 10]. In this study, we compared the long-term clinical outcomes in SIHD patients with and without diabetes who underwent PCI at a large tertiary care center. The objective was to compare, through the use of our prospective PCI registry database, angiographic and procedural characteristics associated with late mortality and morbidity outcomes of DM and non-DM patients in a contemporary PCI practice.

## Materials and methods

A total of 200 patients with SIHD were admitted to the Cardiology Department of Lady Reading Hospital, Peshawar, Pakistan between January 2019 and January 2020 who were symptomatic despite medications or were high risk on non-invasive testing. Data were retrieved from Hospital Management and Information System (HMIS), including socio-demographic characteristics, PCI procedure record, relevant medical history, and in-hospital complications. The patients were followed-up at three months, six months, and one year for data collection regarding mortality and complications. The following cardiovascular risk factors were assessed: hypertension, diabetes mellitus, dyslipidemia, peripheral vascular disease, stroke, prior myocardial infarction, prior revascularization PCI or CABG, smoking, and in-hospital conditions (stent thrombosis, arrhythmia, cardiogenic shock, etc.). Patients who were admitted with acute coronary syndrome and underwent primary or facilitated PCI were excluded. In-hospital outcomes included death, contrast-induced nephropathy, and hemorrhagic complications. One-year outcomes included death, nonfatal myocardial infarction, and revascularization, including target vessel revascularization (TVR) and target lesion revascularization (TLR), as well as outcomes of major adverse cardiovascular events (MACE) as any of the above individual outcomes. Baseline data and outcome data were compared between the two groups. All data were analyzed using SPSS version 23.0 (IBM Corp, Armonk, USA). Binary data were presented by percentage, while qualitative data were presented as mean ± SD. Both groups were compared using a Chi-square test and p ≤ 0.05 was considered significant in all statistics.

## Results

The mean differences and cross-tabulation of baseline and clinical parameters are given in Table 1. The history of CABG was observed to be significantly (p = 0.04)) associated with patients among two groups, that is, DM with SIHD and non-DM with SIHD. The mean age (SD) of the non-diabetic with SIHDs patients was higher (57.4±8.9 years; p = 0.8) than DM patients. Patients with a previous history of CABG were more prevalent in diabetics than nondiabetics (14% vs 4%; p = 0.04 ) All the baseline characteristics were different in the two groups but insignificant statistically.

**Table 1:** Baseline patients Characteristics: CAD: coronary artery disease; CABG: coronary artery bypass grafting; LVEF: left ventricular ejection fraction; SIHD: stable ischemic heart disease

N=200	Diabetic with SIHD, N=100	Non-diabetic with SIHD, N=100	p-value
Age, years (mean±SD)	54.4±8.3	57.4±8.9	0.8
Male	54	74	0.18
Family History of CAD	72	54	0.4
Smoking,	18	26	0.5
Previous history of CABG	14	4	0.04
Hypertension	66	44	0.5
Aspirin	94	88	0.5
Beta blocker	70	76	0.8
Calcium channel b	16	24	0.4
Nitrates	48	38	0.35
Lipid-lowering drug	90	84	0.9
LVEF	52±14	54±16	0.4

Triple vessel disease prevalence was more in DM than in non-DM patients with SIHD, and statistically insignificant. Although both groups had comparable involvement of left anterior descending artery (LAD), left circumflex artery (LCX) involvement was more in diabetics (16% vs 6%; p = 0.03). The number of stents implanted per patients (2.8±0.7 vs 1.9±0.8) was more in DM patients than non-DM patients with SIHD, with most drug-eluting stent (DES) being used in both groups. The angiographic characteristics and procedural characteristics are given in Table 2.

**Table 2:** Immediate Angiographic characteristics and procedural results SIHD: stable ischemic heart disease; LAD: left anterior descending artery; LCX: left circumflex artery; RCA: right coronary artery; LMCA: left main coronary artery; SVG: saphenous vein grafts; DES: drug-eluting stent; BMS: bare-metal stent; PCI: percutaneous coronary intervention

N=200	Diabetic with SIHD, N=100	Non-diabetic with SIHD, N=100	p-value
Single vessel disease	31	34	0.6
Double vessel disease	33	44	0.45
Triple vessel disease	36	22	0.08
Target Vessel			
LAD lesion	58	74	0.6
LCX lesion	16	6	0.03
RCA lesion	16	14	0.46
LMCA or SVG	10	6	0.7
Number of stents implanted per patients	2.8±0.7	1.9±0.8	0.8
Stent Type			
DES Resolute Integrity	23	19	0.40
DES Xience Prime	35	27	0.46
DES Promus Element	32	36	0.46
BMS	10	18	0.38
PCI Type			
Single Vessel PCI	48	30	0.4
Double Vessel PCI	32	38	0.5
Multivessel PCI	20	32	0.7

In-hospital outcomes like contrast-induced nephropathy were found to be significantly different in both groups (5% vs 2%; p = 0.05), similarly, periprocedural myocardial infarction (3% vs 1%; p = 0.05) was also found to be significant between the groups. However, death due to cardiac cause, hyperacute stent thrombosis was slightly higher in the diabetic group, though not statistically significant. The details are given in Table 3.

**Table 3:** In-hospital outcomes MACE: major adverse cardiovascular event; SIHD: stable ischemic heart disease; Hb: hemoglobin

In-hospital outcome MACE, N=200	Diabetic with SIHD, N=100	Non-diabetic with SIHD, N=100	p-value
Death due to cardiovascular cause	2	1	0.06
Periprocedural Myocardial Infarction	3	1	0.04
Hyperacute stent thrombosis	3	2	0.5
Contrast-induced nephropathy	5	2	0.05
Access site Hematoma > 5 cm	3	4	0.3
Hb drop > 5 gm%	2	3	0.5

Among the two groups, five patients were lost to follow up in DM-SIHD group while seven patients in the non-DM with SIHD group. At the end of one year, in terms of MACE, 60 and 70 patients were asymptomatic in the DM-SIHD and non-DM-SIHD groups, respectively. Moreover, the association was noted to be non-significant (p = 0.5). Ischemia-driven revascularization was more common in the diabetic group and was statistically significant. The details are given in Table 4 below. 

**Table 4:** Outcomes associated with 1-year period follow-up MACE: major adverse cardiovascular event; SIHD: stable ischemic heart disease; STEMI: ST-segment elevation myocardial infarction; NSTEMI: non-STEMI; CABG: coronary artery bypass grafting

Follow-up patients	Diabetic with SIHD, N=100	Non-Diabetic with SIHD, N=100	p-value
Lost to follow-up	5	7	0.9
One year MACE			
Death due to cardiovascular cause	3	2	0.6
NSTEMI in the follow up	3	2	0.8
STEMI in the follow up	4	3	0.9
Stent thrombosis acute late	3	4	0.08
In-Stent Restenosis	3	2	0.8
CABG after Re angiography	3	0	
Asymptomatic	60	70	0.5
Medically treated angina	8	7	0.9
Other vessel revascularization	8	3	0.05

## Discussion

DM is a recognized predictor for worse clinical outcomes after percutaneous coronary intervention (PCI) in comparison to non-DM patients. Studies show that DM has an adverse impact on short and long-term clinical outcomes [4-6]. While the global prevalence of DM in 2019 was estimated to be 9.3% (463 million people), 26.3 % of adults of age 19 years and above in Pakistan were living with diabetes, as reported by the National Diabetes Survey of Pakistan [7].

Diabetic patients presenting with myocardial infarction in a significant proportion undergo PCI due to comorbidities and need regular follow up for optimization of medical therapy after PCI. The aim of the present study was to evaluate the incidence of MACE (death, preprocedural myocardial infarction, STEMI/NSTEMI, stent thrombosis late, stent restenosis, or target lesion revascularization) during a follow-up of at least one year in both diabetic and non-diabetic patient with SIHD and the effect of DM on patients who underwent PCI. Based on these parameters, modern-day DESs offered better clinical safety than bare-metal stents (BMS)s and early-generation DESs.

The mean (SD) age in our study was 54.4±8.3 years in DM and 57.4±8.9 years in non-DM patients, which is consistent with previous studies. The occurrence of CAD a decade ago in Pakistan compared to patients from India and Western countries, leading to a loss of productive years of their lives while fighting this disease [8-10]. Regarding the angiographic profile, double vessel disease was the common angiography pattern and LAD was the most commonly involved vessel in both groups, which is consistent with previous studies [8, 11]. The current study shows that DM patients are more at risk of adverse clinical outcomes after PCI, either during the hospital stay or at a one-year follow-up, which is similar to the findings of Zhuo et al. [12]. In our study, stent thrombosis and contrast-induced nephropathy have been found to be higher in DM patients during their hospital stay, but at one year follow-up MACE, followed by stent thrombosis, were more in the non-DM group, which is in line with previous studies [12].

Our study results show that triple vessel disease is more prevalent in the DM group compared to the non-DM group. DM patients with SIHD were prone to more lesions treated per patient and increased adverse events, which is almost similar to the findings reported by Banning et al. [13]. One possible reason may be that vessel diameter and length of lesion predict stent thrombosis in DM patients predisposed to adverse events. An additional possible mechanism could be due to increased platelet aggregations and hyporesponsiveness to clopidogrel and aspirin, resulting in stent thrombosis, which could lead to death directly in several cases [14,15].

Our study results show higher mortality (death), MI (STEMI/NSTEMI), and other vessel revascularization in the DM group during a one-year follow-up after PCI. This finding is in line with Mathew et al. [16]. Similarly, a study by Zhuo et al. [12] also demonstrated worse outcomes in DM patients on follow-up. Kedhi et al. [17] also reported a high number of deaths, MI, and stent thrombosis in the DM group after PCI, thus supporting our findings. Another study reported high mortality in hospitalized patients with DM [18]; in contrast to this study, we have found low mortality in hospitalized PCI patients. Regarding stent thrombosis, it was higher in DM than non-DM patients during the hospital stay and at one-year follow-up in our study, which is almost similar to the findings of Kedhi et al., which reported 3.2% and 1.7% stent thrombosis in DM and non-DM group, respectively **[**17**].** In contrast, in the EVASTENT study, out of 844 patients with DM, 45 patients (5.3%) had stent thrombosis [19]. The small number of stent thrombosis cases in our study may be due to the small sample size.

However, a few studies' results were different from our study's results. Silber et al. reported that not even a single DM patient developed stent thrombosis [20]. Similarly, in the SORT OUT IV trial, no definite stent thrombosis was seen in DM patients treated with everolimus-eluting stents (EES)** **[21]. Another study also reported a lower risk of stent thrombosis in both groups, and the stent thrombosis risk also did not differ by DM status, either with DES or BMS. However, MI and late stent thrombosis incidences were greater in non-DM patients treated with DESs, and only a single patient with DM developed late stent thrombosis [22].

The strength of our study is that it is the first study in Pakistan to look for outcomes in DM patients with SIHD after PCI and the first PCI registry from Lady Reading Hospital, Khyber Pakhtunkhwa, Pakistan. The small sample size is the first limitation of our study. Second, the follow-up time is relatively short, which may not expose the long-term outcomes in DM patients following PCI. Third, the stent size, disease duration of diabetes, diabetes control status, and the insulin use or oral anti-diabetes medications were ignored. Fourth, the dual antiplatelet used duration was not taken into consideration, which is a major limitation of our study. Fifth, the study population is from a single cardiac center and the results of this study cannot be generalized. Hence, further long-term follow-ups and a large sample size need to be studied in the future for better clinical outcomes.

## Conclusions

Our study showed that after undergoing PCI for complex lesions in stable ischemic heart disease both with and without diabetes, the current generation of DES is associated with favorable in-hospital and long-term outcomes. After a mean follow-up of one year, DM and non-DM patients with SIHD showed non-significant differences with regards to MACE. However, ischemia-driven revascularization was more common in DM with SIHD. However, the decision to go for coronary intervention must take into account the clinical angiographic and individual patient profile.
